# Supplementary material for: From sequence to enzyme mechanism using multi-label machine learning
Source: BMC Bioinformatics. 2014 May 19;15:150. doi: 10.1186/1471-2105-15-150 (PMC4229970; doi:10.1186/1471-2105-15-150)
Supplement: Additional file 2 — Java code of ml2db. Additional file ml2db_code.tar.gz contains the Java source code to run the multi-label machine learning experiments and save the results to database. The code’s Javadoc is included. [file 1471-2105-15-150-S2.zip › additional file 2/ml2db/ml2db/doc/mulan/classifier/neural/MMPLearner.html]

MMPLearner


JavaScript is disabled on your browser.


- Overview
- Package
- Class
- Use
- Tree
- Deprecated
- Index
- Help

- Prev Class
- Next Class

- Frames
- No Frames

- All Classes

- Summary:
- Nested |
- Field |
- Constr |
- Method

- Detail:
- Field |
- Constr |
- Method


mulan.classifier.neural

## Class MMPLearner

- java.lang.Object
- - mulan.classifier.MultiLabelLearnerBase
  - - mulan.classifier.neural.MMPLearner

- All Implemented Interfaces:
  :   java.io.Serializable, MultiLabelLearner, weka.core.TechnicalInformationHandler

  ---

    

  ```
  public class MMPLearner
  extends MultiLabelLearnerBase
  ```

  Implementation of Multiclass Multilabel Perceptrons learner. For more information, see

  Version:
  :   2012.02.27

  Author:
  :   Jozef Vilcek

  See Also:
  :   Serialized Form

- - ### Constructor Summary

    Constructors

    Constructor and Description

    `MMPLearner()`

    Default constructor using RankingLoss and uniform update

    `MMPLearner(RankingLossFunction lossMeasure,
    MMPUpdateRuleType modelUpdateRule)`

    Creates a new instance of `MMPLearner`.

    `MMPLearner(RankingLossFunction lossMeasure,
    MMPUpdateRuleType modelUpdateRule,
    long randomnessSeed)`

    Creates a new instance of `MMPLearner`.
  - ### Method Summary

    Methods

    | Modifier and Type | Method and Description |
    | --- | --- |
    | `boolean` | `getConvertNominalToBinary()` Gets a value indication whether conversion of nominal attributes from input data set to binary takes place prior to learning (and respectively making a prediction). |
    | `weka.core.TechnicalInformation` | `getTechnicalInformation()` Returns an instance of a TechnicalInformation object, containing detailed information about the technical background of this class, e.g., paper reference or book this class is based on. |
    | `int` | `getTrainingEpochs()` Gets number of training epochs. |
    | `java.lang.String` | `globalInfo()` Returns a string describing the multi-label learner. |
    | `boolean` | `isUpdatable()` Returns value indicating if learner is updatable, so if learner is able to perform on-line learning. |
    | `MultiLabelOutput` | `makePredictionInternal(weka.core.Instance instance)` Learner specific implementation for predicting on specified data based on trained model. |
    | `void` | `setConvertNominalToBinary(boolean convert)` Sets whether nominal attributes from input data set has to be converted to binary prior to learning (and respectively making a prediction). |
    | `void` | `setTrainingEpochs(int epochs)` Sets the number of training epochs. |

    - ### Methods inherited from class mulan.classifier.MultiLabelLearnerBase

      `build, getDebug, makeCopy, makePrediction, setDebug`
    - ### Methods inherited from class java.lang.Object

      `equals, getClass, hashCode, notify, notifyAll, toString, wait, wait, wait`

- - ### Constructor Detail


    - #### MMPLearner

      ```
      public MMPLearner()
      ```

      Default constructor using RankingLoss and uniform update


    - #### MMPLearner

      ```
      public MMPLearner(RankingLossFunction lossMeasure,
                MMPUpdateRuleType modelUpdateRule)
      ```

      Creates a new instance of `MMPLearner`.

      Parameters:
      :   `lossMeasure` - the loss measure to be used when judging
          ranking performance in learning process
      :   `modelUpdateRule` -


    - #### MMPLearner

      ```
      public MMPLearner(RankingLossFunction lossMeasure,
                MMPUpdateRuleType modelUpdateRule,
                long randomnessSeed)
      ```

      Creates a new instance of `MMPLearner`.

      Parameters:
      :   `lossMeasure` - the loss measure to be used when judging
          ranking performance in learning process
      :   `modelUpdateRule` -
      :   `randomnessSeed` - the seed value for pseudo-random generator
  - ### Method Detail


    - #### setTrainingEpochs

      ```
      public void setTrainingEpochs(int epochs)
      ```

      Sets the number of training epochs. Must be greater than 0.  
      Default value is 1.

      Parameters:
      :   `epochs` - the number of training epochs

      Throws:
      :   `java.lang.IllegalArgumentException` - if passed value is invalid


    - #### getTrainingEpochs

      ```
      public int getTrainingEpochs()
      ```

      Gets number of training epochs.
      Default value is 1.

      Returns:
      :   training epochs


    - #### setConvertNominalToBinary

      ```
      public void setConvertNominalToBinary(boolean convert)
      ```

      Sets whether nominal attributes from input data set has to be converted to binary
      prior to learning (and respectively making a prediction).

      Parameters:
      :   `convert` - flag indicating whether conversion should take place


    - #### getConvertNominalToBinary

      ```
      public boolean getConvertNominalToBinary()
      ```

      Gets a value indication whether conversion of nominal attributes from input data
      set to binary takes place prior to learning (and respectively making a prediction).

      Returns:
      :   value indication whether conversion takes place


    - #### isUpdatable

      ```
      public boolean isUpdatable()
      ```

      **Description copied from interface: `MultiLabelLearner`**

      Returns value indicating if learner is updatable, so if learner is able to
      perform on-line learning. The fact if learner is updatable or not influences
      the behavior of `MultiLabelLearner.build(MultiLabelInstances)` method.  
      When `false` is returned, each call of the
      `MultiLabelLearner.build(MultiLabelInstances)` will initialize the learner from
      the scratch, removing any potential knowledge built by previously entered training data.
      When `true` is returned, then on the first call of the
      `MultiLabelLearner.build(MultiLabelInstances)` the learner is initialized
      with the passed training data. All other calls contribute to the existing learner's
      model with new data.

      **Specified by:**
      :   `isUpdatable` in interface `MultiLabelLearner`

      **Overrides:**
      :   `isUpdatable` in class `MultiLabelLearnerBase`

      Returns:
      :   `true` if learner is updatable (on-line), `false` otherwise.


    - #### makePredictionInternal

      ```
      public MultiLabelOutput makePredictionInternal(weka.core.Instance instance)
                                              throws InvalidDataException
      ```

      **Description copied from class: `MultiLabelLearnerBase`**

      Learner specific implementation for predicting on specified data based on trained model.
      This method is called from `MultiLabelLearnerBase.makePrediction(weka.core.Instance)` which guards for model
      initialization and apply common handling/behavior.

      Parameters:
      :   `instance` - the data instance to predict on

      Returns:
      :   the output of the learner for the given instance

      Throws:
      :   `InvalidDataException` - if specified instance data is invalid and can not be processed by the learner


    - #### getTechnicalInformation

      ```
      public weka.core.TechnicalInformation getTechnicalInformation()
      ```

      **Description copied from class: `MultiLabelLearnerBase`**

      Returns an instance of a TechnicalInformation object, containing detailed
      information about the technical background of this class, e.g., paper
      reference or book this class is based on.

      **Specified by:**
      :   `getTechnicalInformation` in interface `weka.core.TechnicalInformationHandler`

      **Specified by:**
      :   `getTechnicalInformation` in class `MultiLabelLearnerBase`

      Returns:
      :   the technical information about this class


    - #### globalInfo

      ```
      public java.lang.String globalInfo()
      ```

      **Description copied from class: `MultiLabelLearnerBase`**

      Returns a string describing the multi-label learner.

      **Specified by:**
      :   `globalInfo` in class `MultiLabelLearnerBase`

      Returns:
      :   a description suitable for displaying in a future gui


- Overview
- Package
- Class
- Use
- Tree
- Deprecated
- Index
- Help

- Prev Class
- Next Class

- Frames
- No Frames

- All Classes

- Summary:
- Nested |
- Field |
- Constr |
- Method

- Detail:
- Field |
- Constr |
- Method
